# Nontargeted metabolomics analysis of potential biomarkers for patients with chronic ischemic stroke in extremely cold rural regions: An exploratory case-control study

**DOI:** 10.1371/journal.pone.0341966

**Published:** 2026-02-20

**Authors:** Li Bai, Zhongyuan Li, Jie Ge, Xiaolei Yang, Haifeng Xue, Baokui Qi, Liran Cui, Kejia Zhu, Yu Cheng, Xueyan Qian, Yuehui Jia, Hongjie Li, Jiping Li, Gang Li, Jiyuan Li, Shuli Ma, Yufei Liu, Hong Chao

**Affiliations:** 1 Qiqihar Medical University, School of Public Health, Qiqihar, Heilongjiang, China; 2 Qiqihar Medical University Affiliated First Hospital, Qiqihar, Heilongjiang, China; 3 Heilongjiang Center for Disease Control and Prevention, Harbin, Heilongjiang, China; Southern Illinois University Carbondale, UNITED STATES MINOR OUTLYING ISLANDS

## Abstract

**Objective:**

Chronic ischemic stroke (CIS) is a serious cardiovascular event, closely related to genetic and environmental factors in its occurrence and development. This study aims to reveal the potential impact of extreme cold environments on patients with CIS by comparing the metabolic characteristics of different populations, and to explore potential biomarkers and metabolic differences between Daur and Han CIS patients.

**Methods:**

A total of 32 patients with CIS and 32 matched by age, sex and race healthy controls were included in this study. Their demographic data were collected and clinical indicators were measured. The potentially differential expressed metabolites (DEMs) associated with CIS were identified using liquid chromatography-mass spectrometry (LC-MS/MS). Statistical analysis was performed using SPSS 22.0 and MetaboAnalyst 6.0.

**Results:**

1. There were statistically significant differences in urea nitrogen (*P* = 0.006), history of hypertension (*P* = 0.006), and history of diabetes (*P* = 0.046) between the patients with CIS and healthy controls. The association between a history of hypertension and CIS had an odds ratio of 4.023 (95% confidence interval 1.294–12.513). 2. Nontargeted metabolomics analysis identified 29 potential DEMs associated with CIS, which were further analyzed. A receiver operating characteristic (ROC) curve demonstrated the moderate performance (AUC = 0.750, accuracy rate = 66.5%) of these potential DEMs in identifying patients with CIS. The galactose metabolic pathway, as well as cysteine and methionine metabolism, were significantly different between the groups (*P* = 0.006 and *P* = 0.009, respectively). 3. Metabolomic analysis comparing Daur and Han patients with CIS identified 37 potential DEMs. A ROC curve for these potential DEMs was constructed (AUC = 0.942, accuracy rate = 87.4%). The metabolic pathways of cysteine and methionine metabolism, as well as arginine and proline metabolism, were significantly different between Daur and Han patients with CIS (*P* = 0.021 and *P* = 0.023, respectively).

**Conclusion:**

This exploratory study identified potential DEMs associated with CIS in populations from extremely cold rural regions and within the Daur ethnic subgroup. A preliminary ROC model constructed using these DEMs indicated their potential diagnostic value for CIS, though this requires further validation in large-scale, independent cohort studies.

## Introduction

Ischemic stroke (IS) refers to ischemic necrosis of brain tissue caused by blood supply disorders to the brain, resulting in ischemia and hypoxia. It has an acute onset and causes severe damage [1]. IS has a complex pathophysiology, which mainly includes cell necrosis and apoptosis, oxidative stress, excitatory injury, inflammation, etc, [2]. Accumulating evidence suggests that cold exposure is, to some extent, a potential risk factor for IS. Several potential mechanisms have been mentioned in recent studies. For example, the seasonal and temperature variability of cerebrovascular risk factors (hypertension, hyperglycemia, hyperlipidemia, and atrial fibrillation) may be involved. The temperature variability of blood pressure, blood glucose and blood lipids and their poor control rates in winter are among the reasons for the peak incidence of IS coinciding with this reason [3]. Moreover, a low-temperature environment affects resting energy expenditure (REE). Previous studies have indicated that REE changes upon long-term exposure to different temperatures [4,5]. During cerebral ischemia, parts of the brain lack oxygen and nutrients and are severely damaged by the resulting metabolic and cellular disorders.

In 2020, there were 17.8 million stroke patients aged 40 years and above in China [6]. The crude mortality rate for stroke among rural residents was 164.77 per 100,000 people [7]. IS is the primary cause of disability in developing countries and one of the most common causes of death globally [8]. Approximately half of survivors face long-term disabilities, imposing a heavy burden on individuals, families and society [9]. The stages of IS can be defined as hyperacute (<6 h), acute (6–72 h), subacute (>72 h), and chronic stages (>6 weeks). Each stage has its own distinct pathological features, especially in regard to the effects on the blood-brain barrier [10]. At present, although the mainstream therapies of intravenous thrombolysis and endovascular thrombectomy can effectively improve the prognosis of some patients, there are still inherent drawbacks, such as limited beneficiary groups, potential side effects, and being limited by strict therapeutic time window requirements [11–13]. Many patients do not seek or receive timely treatment, resulting in obvious brain damage and disabilities. Timely and accurate diagnosis and reperfusion decision-making are the key factors in reducing the disability rate and severity of IS. Therefore, on the basis of in-depth analysis of the pathological staging characteristics of IS, more universal diagnosis and treatment plans are urgently needed, and the application potential of metabolomics technology in disease classification, diagnosis and individualized treatment deserves additional attention.

Metabolomics has unique advantages in the discovery of disease biomarkers and the analysis of pathological mechanisms [14,15]. Nontargeted metabolomics, as the core method of systems biology research, detects all metabolites in a sample without bias and constructs a global metabolic map. With the help of mass spectrometry database comparisons, novel metabolites can be revealed [16,17]. Although metabolomics is still in its infancy in the research on IS, its wide application in patients with IS can provide new insights for predicting the risk of stroke. These dynamically changing metabolites not only are significantly correlated with the degree of neurological deficits, but can also clarify the molecular basis of clinical phenotypes through metabolic pathway enrichment analysis [18,19].

This study explored untargeted metabolomics to profile differential metabolites in CIS patients from extremely cold rural regions, seeking to characterize the associations between metabolites and prognosis. This enables nontargeted metabolomics not only to reveal the core mechanisms of IS, such as blood pressure, anti-inflammatory effects and oxidative stress, but also to propose novel molecular hypotheses for precision treatment and recurrence prevention. Given its exploratory design and limited sample size, we explicitly position this work as a pilot investigation. All identified metabolic signatures necessitate validation in larger, independent cohorts.

## Materials and methods

### Study design and participants

This study selected 32 patients with CIS who met the diagnostic criteria and were recruited from the township health centers of Fulaerji District and Meilisi District in Qiqihar city, Heilongjiang Province, from March 2024 to March 2025 as the case group, and 32 healthy individuals matched by age (±1 year), sex (exact match) and race (exact match) at a 1:1 ratio were selected as the control group. Our sample size is relatively small, which may limit the statistical power to results. The research protocol was reviewed by the Ethics Committee of Qiqihar Medical University (Approval Number: (Qi) Lun Shen [2021] No. 76), and all participants signed a written informed consent form.

Inclusion criteria. Patients in the case group were diagnosed with CIS by brain magnetic resonance diffusion-weighted imaging and voluntarily signed the informed consent form. There was no history of cerebrovascular disease. No anticoagulant or antiplatelet therapy was given within the past three months.

Exclusion criteria. Patients with organic neurological diseases such as Alzheimer’s disease and brain tumors, severe liver and kidney dysfunction, hematological diseases or a history of using immunosuppressants within the past 6 months were excluded.

### Data collection

A professionally trained survey team conducted face-to-face surveys using standardized questionnaires to collect the following data. Basic information, including social and economic conditions such as age, sex, ethnic demographic and sociological characteristics, educational level, marital status, occupation type and annual household income, lifestyle assessment such as smoking history, alcohol and tea drinking habits, dietary structure, exercise intensity and sleep quality, and personal chronic disease history, etc., was collected. Physiological parameters, including height, weight, waist circumference, hip circumference, lung capacity and blood pressure were collected. The laboratory test data collected included routine blood parameters, fasting blood glycose level, glycated hemoglobin level, four lipid parameters (total cholesterol, triglycerides, high-density lipoprotein, and low-density lipoprotein levels), and liver and kidney function indicators (ALT, AST, BUN, and Cr levels).

Diagnostic Criteria and Definitions. Hypertension was defined as one of the following conditions: a confirmed medical history from a medical institution, or systolic blood pressure ≥140 mmHg and/or a diastolic blood pressure ≥ 90 mmHg measured three times at rest, or the regular use of antihypertensive drugs for ≥ 1 month. Diabetes was defined as one of the following conditions: a clear diagnosis recorded in the endocrinology department, or FPG ≥ 7.0 mmol/L or HbA1c ≥ 6.5%, or continuous use of hypoglycemic drugs or insulin treatment. Cardiovascular and cerebrovascular diseases were defined as an IS or coronary heart disease diagnosis verified through the hospital medical record system. Smoking refers to the intake of one or more cigarettes per day for a continuous or cumulative period of six months. Alcohol consumption refers to the intake of ethanol twice or more per week for a period of 12 months or more. Regular exercise refers to 30 minutes or more of physical activity each time, three or more times a week.

### Instruments and reagents

Experimental Instruments. An ultrahigh-performance liquid chromatograph (Vanquish, Thermo Fisher Scientific), a high-resolution mass spectrometer (Orbitrap Exploris 120, Thermo Fisher Scientific), a low-temperature high-speed centrifuge (Heraeus Fresco 17, Thermo Fisher Scientific), an analytical balance (BSA124S-CW, Sartorius), an ultrasonic instrument (PS-60AL, Shenzhen Redbang Electronics Co., LTD.), and a freeze dryer (LGJ-10C) (Sihuan Furui Keyi Technology Development Co., LTD.) were used in this study.

Experimental Reagents. Methanol (CNW Technologies, LC-MS grade), acetonitrile (CNW Technologies, LC-MS grade), ammonium acetate (Sigma-Aldrich, LC-MS grade), ammonia water (CNW Technologies) (LC-MS grade), acetic acid (Sigma-Aldrich, LC-MS grade), and isopropanol (CNW Technologies, LC-MS grade) were used.

### The biological specimen bank

Whole blood was collected through peripheral veins from the subjects in the morning after they fasted for 8 hours. The blood was distributed into four tubes, including two 5 ml/-tube EDTA anticoagulant tubes and two 2 ml/-tube separator tubes. One blood sample was transferred to the Laboratory Center of the Affiliated Hospital of Qiqihar Medical University under real-time temperature monitoring at 2–8 °C for routine blood tests, liver and kidney function tests, glycolipid metabolism indicators, etc. Another sample was processed in the local laboratory, and plasma separation was completed within 2 hours. The serum was separated at 3000 rpm for 15 minutes at 4 °C, aliquoted into 2 ml cryotubes, rapidly frozen and stored in an ultralow temperature freezer at −80 °C.

### Nontargeted metabolomics

One hundred microliter of serum was transferred to a 96-well protein precipitation plate, and 400 μl of precooled extraction solvent (methanol: acetonitrile = 1:1 (v/v), with an isotope internal standard) was added. The mixture was shaken and mixed well (750 rpm, 5 minutes), allowed to stand for sedimentation for 5 minutes, and filtered, and the filtrate was collected for testing. Equal amounts of the supernatants from each sample were mixed to form QC samples for machine testing.

For the determination of polar metabolites, a Vanquish (Thermo Fisher Scientific) ultrahigh-performance liquid chromatograph was used. The target compound was chromatographically separated on a Waters ACQUITY UPLC BEH amide (2.1 mm × 50 mm, 1.7 μm) liquid chromatography column. The Orbitrap Exploris 120 mass spectrometer is capable of conducting mass spectrometry data acquisition under the control of its software (Xcalibur, version 4.4; Thermo). Phase A of liquid chromatography was the aqueous phase, containing 25 mmol/L ammonium acetate and 25 mmol/L ammonia water, and phase B was acetonitrile. The sample tray temperature was 4 °C, and the injection volume was 2 μL. Mass spectrometry data were acquired in information-dependent acquisition (IDA) mode, with a full scan MS resolution of 60,000 and MS/MS resolution of 15,000. The electrospray ionization (ESI) source parameters were as follows: sheath gas flow rate 50 Arb, auxiliary gas flow rate 15 Arb, capillary temperature 320°C, collision energy 20/30/40, spray voltage in positive ion mode 3.8 kV, and −3.4 kV in negative ion mode.

### Data processing and statistical analysis

Statistical analysis was performed using SPSS 22.0 and MetaboAnalyst 6.0. Continuous variables (such as age and biochemical indicators) are expressed as $\;\overset{―}{x}\pm s$, and t-tests or ANOVA were used for comparisons between groups. Categorical variables (sex, smoking, drinking, etc.) are expressed as frequencies and composition ratios (n/%), and the chi-square test was used for comparisons between groups. Correlation analysis was performed using Spearman correlation analysis. Statistical analysis was conducted using two-sided tests. Epidemiological data were considered statistically significant when *P* < 0.05. The charts were generated using GraphPad Prism 10.0. The ROC curves of the differentially abundant metabolites and the multivariate prediction model were generated, and the area under the curve (AUC) was calculated.

Nontargeted metabolomics data processing. The data collected by ultra-high-performance liquid chromatography (UHPLC) were imported into ProteoWizard software for peak matching and peak extraction and then imported into EZinfo software for statistical analysis of the identified metabolites to construct a data matrix of the mass-charge ratio, retention time and peak area. Missing values caused by extremely low sample substance content will be imputed using half of the minimum value. Metabolite identification levels were annotated to the Metabolomics Standards Initiative (MSI) guidelines, and list the specific confidence levels of metabolites in S1 Table. All untargeted metabolomic data used in this publication have been deposited to the EMBL-EBI MetaboLights database with the identifier MTBLS13631. The complete data set can be accessed at https://www.ebi.ac.uk/metabolights/MTBLS13631.

The differences between groups were detected by using the orthogonal partial least squares discriminant analysis (OPLS-DA) multivariate statistical method. To verify the stability of the model, 200 permutation tests were conducted. The variable importance projection (VIP) value of each metabolite was determined through multiple cross-validations. Student’s t-test was used to evaluate the statistical significance of inter-group differences (*P*-value). Metabolites with VIP ≥ 1, and *P* < 0.05 were identified as potential DEMs. The identification of potential differential metabolites requires meeting the following criteria. It was included in the HMDB database and confirmed as an endogenous substance (i.e., excluding the parent drug and its metabolites). Pathway enrichment analysis of these differentially abundant metabolites was performed using the Kyoto Encyclopedia of Genes and Genomes (KEGG) database. The ability of the candidate metabolites to distinguish CIS was analyzed using multivariate ROC curves, and the AUC was calculated through Monte Carlo cross-validation (200 iterations).

## Results

### Baseline characteristics of the study population

The baseline characteristics of 32 patients with CIS and 32 healthy controls were matched 1:1 by age, sex and race (Table 1). Compared with those of healthy controls, the demographic characteristics (education level, marital status, education and household income, etc.), living habits (smoking, drinking alcohol, drinking tea, dietary structure, exercise intensity and sleep quality), physiological indicators (BMI, waist-hip ratio, systolic blood pressure, diastolic blood pressure, vital capacity, body fat percentage, etc.), blood parameters (white blood cell count, hemoglobin, platelets, alanine aminotransferase, aspartate aminotransferase, urea, uric acid), and metabolic indicators (total cholesterol, triglycerides, high-density lipoprotein cholesterol, low-density lipoprotein cholesterol) of the CIS group, were not significantly different (*P* > 0.05). However, in terms of urea nitrogen, history of hypertension and history of diabetes, the differences were statistically significant (*P* = 0.006, *P* = 0.006, and *P* = 0.046, respectively). The results of multivariate logistic regression analysis revealed that a previous history of hypertension was a risk factor for CIS, with an OR 95%CI of 4.023 (1.294,12.513) (Table 2).

**Table 1:** Baseline characteristics of CIS patients and healthy controls.

Basic Information	CIS group (n = 32)		HC group (n = 32)		Statistic/*P* value
	Daur (n = 16)	Han (n = 16)	Daur (n = 16)	Han (n = 16)	
**Demographic Characteristics**					
Sex (Male/Female)	7/9	8/8	7/9	8/8	0.251/0.969
Age (years)	65.75 ± 7.01	66.38 ± 7.51	65.88 ± 6.48	66.13 ± 7.27	0.550/0.929
Household income (ten thousand yuan, n/%)					3.096/0.797
≤ 3	13(81.25)	15(93.75)	14(87.50)	12(75.00)	
3-4.99	1(6.25)	1(6.25)	1(6.25)	2(12.50)	
≥ 5	2(12.50)	0(0.00)	1(6.25)	1(6.25)	
Marital status					5.762/0.450
Married	12	12	14	14	
Unmarried	0	0	1	0	
Divorced or widowed	4	4	1	2	8.303/0.217
Education					
Elementary school and below	11	12	10	12	
Middle school	5	4	6	2	
High school and above	0	0	0	2	6.554/0.364
Dietary structure					
Primarily vegetables	8	9	4	4	
Primarily meat	2	2	2	4	
Balanced mix of meat and vegetables	6	5	10	8	
Dairy product intake	28.57(16.08, 167.88)	15.72(0.17, 71.43)	50(0.69, 138.40)	0(0, 55.24)	−1.246/0.213
Smoking (n/%)	5(31.25)	5(31.25)	5(31.25)	4(25.00)	0.225/0.974
Drinking alcohol (n/%)	8(50.00)	6(37.50)	8(50.00)	7(43.75)	0.694/0.875
Drinking tea (n/%)	4(25.00)	3(18.75)	5(31.25)	4(25.00)	0.667/0.881
Physical activity (n/%)	14(87.50)	13(81.25)	14(87.50)	15(93.75)	1.143/0.767
Quality sleep (n/%)	3(18.75)	4(25.00)	5(31.25)	5(31.25)	0.881/0.830
Hypertension (n/%)	8(50.00)	13(81.25)	4(25.00)	5(31.25)	12.298/0.006
Diabetes (n/%)	2(12.50)	5(31.25)	1(6.25)	0(0.00)	8.000/0.046
Abnormal lipid levels (n/%)	7(43.75)	4(25.00)	6(37.50)	5(31.25)	1.385/0.709
**Physical Measurements**					
BMI (kg/m^2^)	26.61 ± 1.88	26.41 ± 3.65	26.37 ± 4.41	24.93 ± 3.89	0.743/0.531
Waist-to-hip ratio (WHR)	0.96 ± 0.04	0.99 ± 0.20	0.95 ± 0.06	0.96 ± 0.07	0.407/0.748
Body fat percentage (%)	33.97 ± 3.95	29.81 ± 6.21	31.29 ± 7.15	28.29 ± 8.16	2.174/0.100
Vital capacity	2419.31 ± 590.89	3156.03 ± 920.41	2946.22 ± 904.05	2850.20 ± 859.55	2.234/0.093
SBP (mmHg)	141.44 ± 16.09	157.44 ± 23.68	151.75 ± 22.95	159.00 ± 16.43	2.385/0.078
DBP (mmHg)	82.00 ± 9.00	85.81 ± 18.52	84.69 ± 16.19	85.56 ± 12.68	0.230/0.875
**Laboratory Data**					
WBC (10^9^/L)	6.44 ± 0.93	6.00 ± 0.81	5.67 ± 2.19	5.41 ± 1.38	1.540/0.213
Hb (g/L)	141.31 ± 7.45	148.06 ± 21.59	142.00 ± 10.68	143.00 ± 13.32	0.736/0.535
PLT (10^9^/L)	242.56 ± 42.28	215.63 ± 60.40	227.63 ± 66.76	238.50 ± 45.96	0.778/0.511
ALT (U/L)	21.06 ± 3.99	20.00 ± 7.04	17.94 ± 5.52	21.00 ± 11.65	0.563/0.642
AST (U/L)	15.06 ± 2.84	17.93 ± 5.56	17.63 ± 5.29	19.13 ± 7.33	1.522/0.211
BUN (mmol/L)	6.59 ± 1.39	7.54 ± 1.72	6.29 ± 1.23	7.93 ± 1.44	4.525/0.006
UA (µmol/L)	320.00 ± −36.66	310.25 ± 83.47	340.13 ± 87.92	350.56 ± 87.83	0.912/0.441
FBG (mmol/L)	5.91 ± 1.47	6.85 ± 2.85	5.23 ± 0.84	5.89 ± 1.69	2.040/0.118
TG (mmol/L)	1.79 ± 0.71	1.41 ± 0.66	1.99 ± 1.04	1.40 ± 1.01	1.734/0.170
TC (mmol/L)	4.81 ± 1.31	5.22 ± 0.85	5.31 ± 1.09	5.90 ± 1.43	2.277/0.089
HDL-C (mmol/L)	1.07 ± 0.11	1.15 ± 0.25	1.18 ± 0.31	1.14 ± 0.25	0.595/0.620
LDL-C (mmol/L)	2.12 ± 0.45	2.16 ± 0.21	2.37 ± 0.39	2.30 ± 0.39	1.169/0.329

Note: CIS: Chronic ischemic stroke; HC: Healthy control; WBC: White blood cell; Hb: Hemoglobin; PLT: Platelet; ALT: Alanine aminotransferase; AST: Aspartate aminotransferase; BUN: Blood urea nitrogen; UA: Uric acid; TC: Total cholesterol; TG: Triglycerides; HDL-C: High-density lipoprotein cholesterol; LDL-C: Low-density lipoprotein cholesterol; SBP: Systolic blood pressure; DBP: Diastolic blood pressure.

**Table 2:** Logistic regression analysis of factors associated with CIS in extremely cold rural regions.

Variable	β	SE	Wald	P value	OR	95%CI
BUN (mmol/L)	−0.041	0.187	0.048	0.827	0.960	0.666 ~ 1.385
Hypertension (n/%)	1.392	0.579	5.782	0.016	4.023	1.294 ~ 12.513
Diabetes (n/%)	1.598	1.150	1.929	0.165	4.943	0.518 ~ 47.118

### Statistical analysis of nontargeted metabolomics in patients with CIS and healthy controls

**Fig 1:**
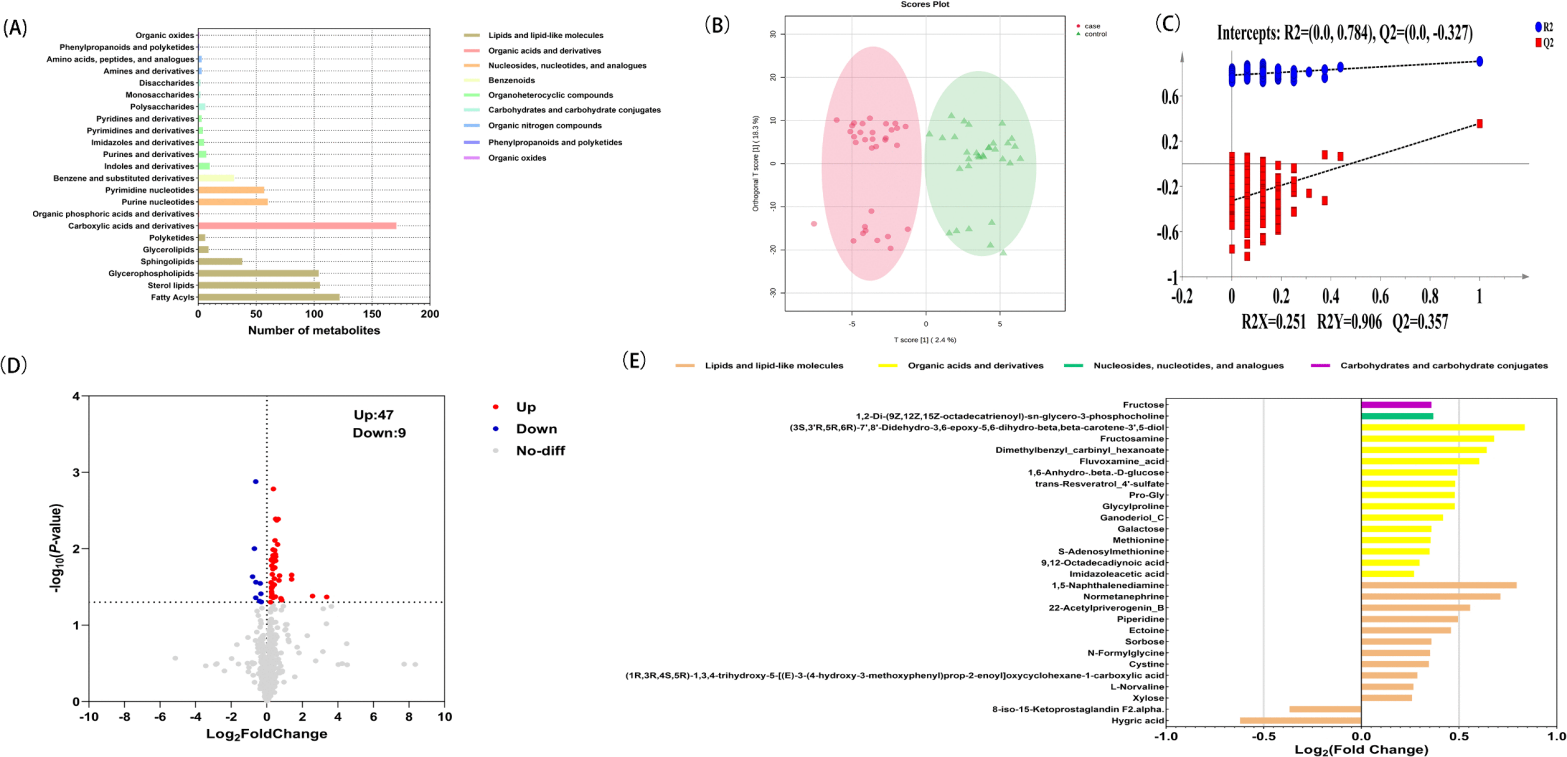
Characteristic metabolites in the serum of CIS patients. **(A)** Bar chart of metabolite classification. The y-axis represents the categories of metabolites, and the x-axis represents the number of metabolites. **(B)** OPLS-DA of serum metabolites. **(C)** Permutation test. **(D)** Volcano plot of DEMs of CIS vs. healthy controls. **(E)** Fold changes in 29 potential DEMs of CIS vs. healthy controls.

Screening of potential DEMs. A total of 1,308 serum metabolite characteristics were detected, among which 751 were annotated by the HMDB. These metabolites can be classified into four categories: 384 lipids and lipid-like molecules, 172 organic acids and derivatives, 117 nucleosides, nucleotides, and analogues, 31 benzenoids, 29 organoheterocyclic compounds, 10 carbohydrates and carbohydrate conjugates, 6 organic nitrogen compounds, 1 phenylpropanoids and polyketides and 1 organic oxides (Fig 1A). The internal standard response stability and the correlation analysis of QC samples showed good stability and repeatability, which can support subsequent analysis (S2 Table and S1 Fig.). The OPLS-DA score map was constructed using the identified nontargeted metabolites to characterize the metabolic characteristics (Fig 1B). The OPLS-DA model revealed significant cluster separation between the CIS group and the healthy control group. The cumulative R2Y is 0.906, and Q2 is 0.357, indicating a good fit. The slope of the fitted straight line is positive, and the intercept of Q2 on the Y-axis is −0.327. In the permutation test, the Q2 intercept being less than zero indicates that the original model’s predictive ability is better than of random model, indicating that there is no overfitting phenomenon in the OPLS-DA model (Fig 1C). Metabolites that met the criteria of VIP ≥ 1 and *P* < 0.05 were classified as potential DEMs. The volcano plot shows 56 DEMs, including 47 that are upregulated and 9 that are downregulated (Fig 1D). Exclude 22 substances not present in the HMDB database and 5 drug prototype components and their metabolites. The remaining 29 metabolites were further analyzed, among which 27 were upregulated and 2 were downregulated (Fig 1E and S3 Table).

**Fig 2:**
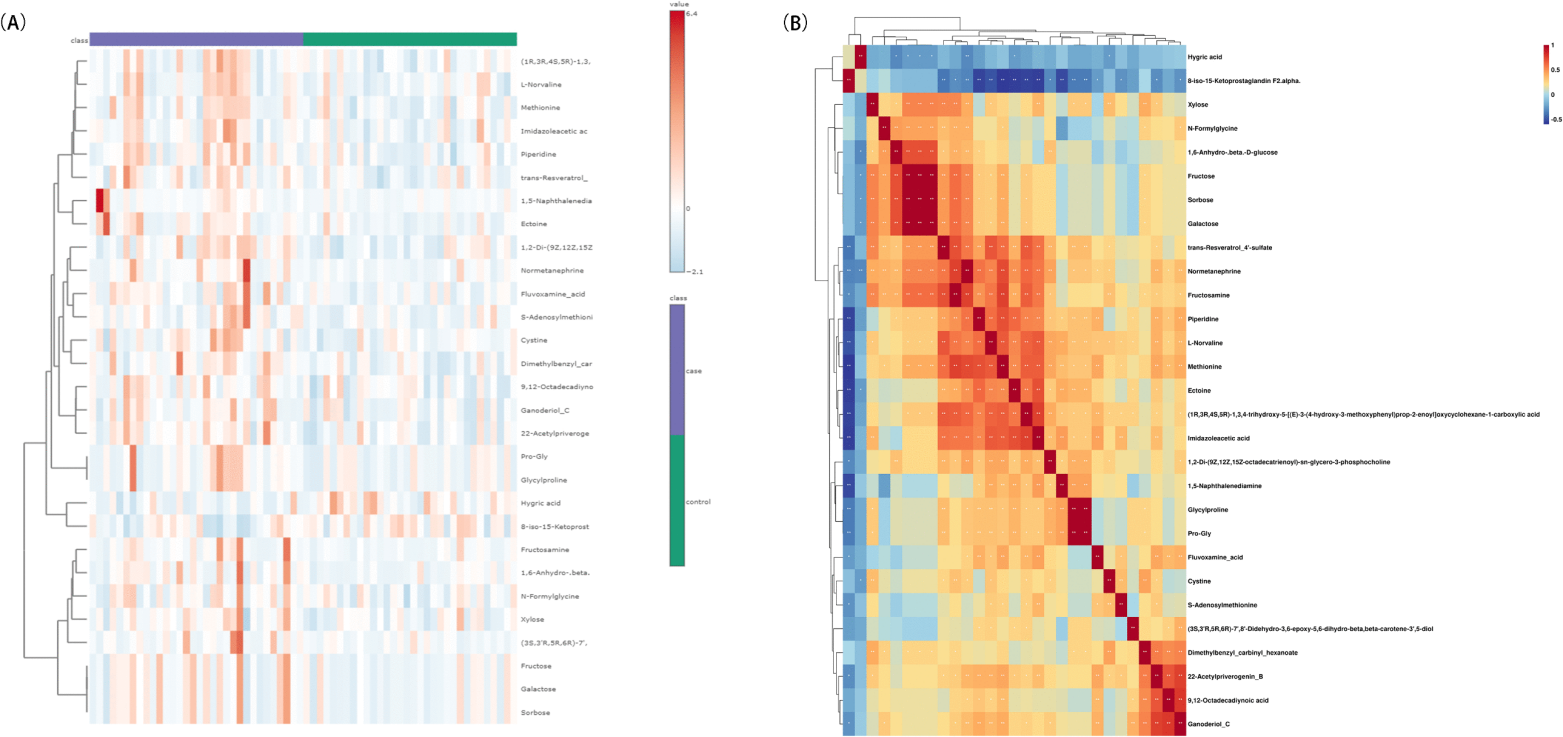
Correlation heatmaps of potential DEMs. **(A)** Clustered heatmap of potential DEMs. **(B)** Clustered heatmap of Spearman correlation coefficients.

Cluster analysis. The clustering heatmap revealed the ability of 29 potential DEMs to distinguish between CIS patients and healthy controls (Fig 2A). Spearman correlation analysis revealed varying degrees of correlation among the expression levels of these 29 potential DEMs (Fig 2B).

**Fig 3:**
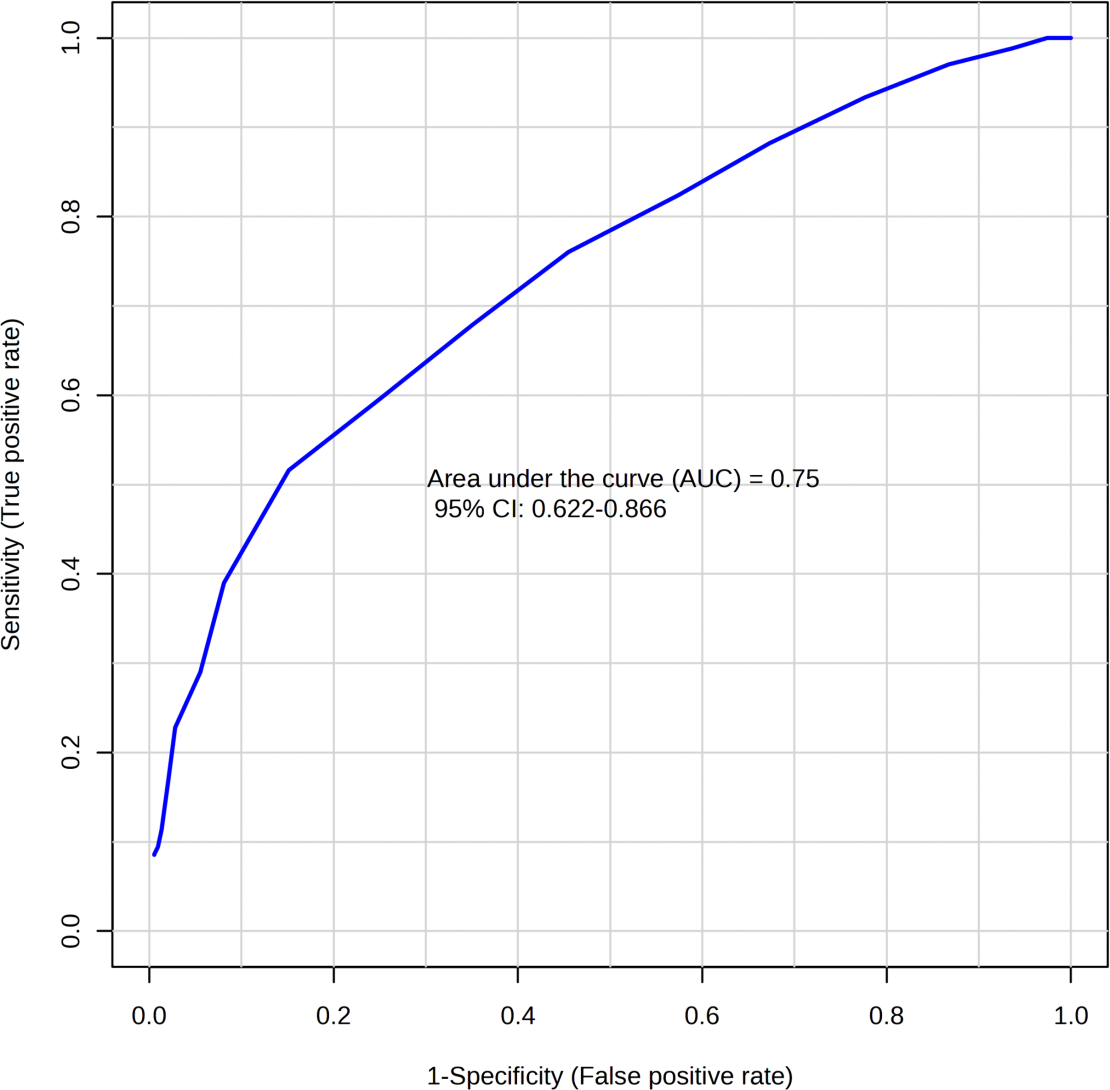
ROC curves of serum DEMs for distinguishing between CIS patients and healthy controls.

ROC curve analysis. Multiple exploratory ROC curves were used to assess the sensitivity and specificity of various metabolites to distinguish between CIS and controls. A multivariate algorithm was established using the metabolite classification method, which is based on PLS-DA, and the feature ranking method, which is built into PLS-DA. The area under the ROC curve (AUC) and 95% CI for the potential DEMs between CIS patients and healthy controls were 0.750(0.622–0.866), with an accuracy of 66.5% (Fig 3). This confirms that ROC analysis demonstrated moderate statistical significance in distinguishing between CIS and healthy controls, but the value of AUC is insufficient to support these metabolites as independent diagnostic tools.

**Fig 4:**
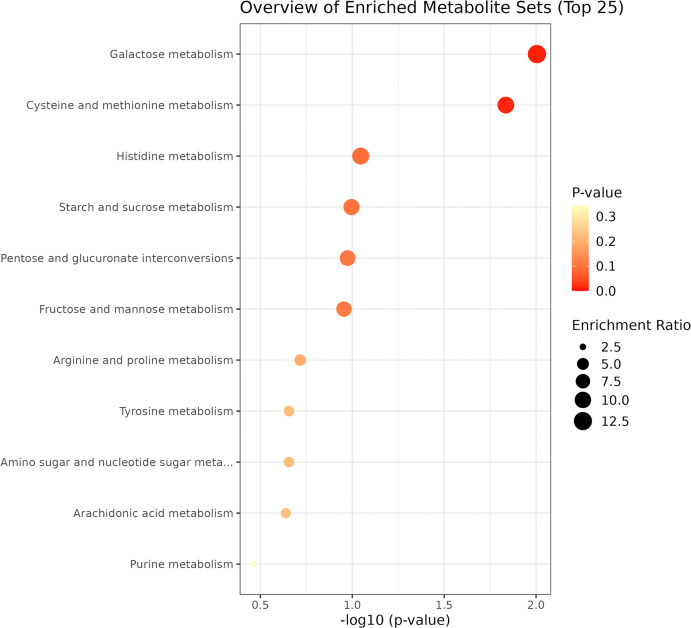
Enrichment analysis of DEMs in CIS patients on the basis of KEGG data.

Pathway enrichment analysis. Twenty-nine potential DEMs were introduced into the KEGG database for metabolic pathway analysis. The results revealed significant differences in the galactose metabolism pathway (*P* = 0.006, expected value = 0.123) and cysteine and methionine metabolism pathway (*P* = 0.009, expected value = 0.150) between CIS patients and healthy controls. This pathway is significantly enriched in CIS due to energy compensation (Fig 4).

### Statistical analysis of nontargeted metabolomics in Daur and Han patients with CIS

**Fig 5:**
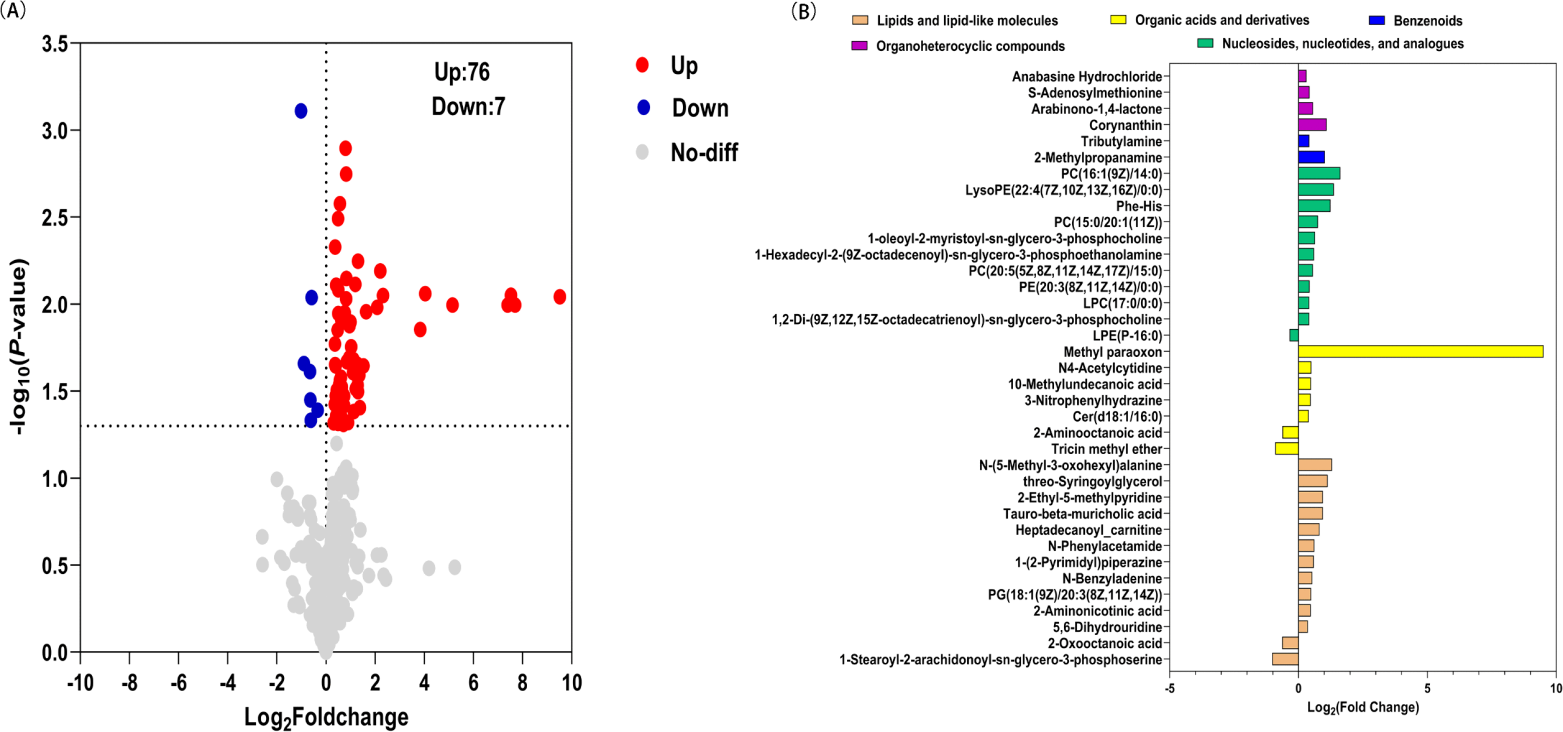
DEMs in the serum of Daur vs. Han patients with CIS. **(A)** Volcano plot of DEMs of the Daur vs. Han patients with CIS. **(B)** Fold changes in 37 DEMs of Daur vs. Han patients with CIS.

Screening of potential DEMs. Metabolites were classified as potential DEMs if they met the criteria of VIP ≥ 1and *P* < 0.05. A volcano plot revealed 83 potential DEMs, including 76 that were upregulated features and 7 that were downregulated (Fig 5A). This study identified and annotated the 83 DEMs using HMDB database. After removing 35 substances not present in the HMDB database and 11 drug prototype components and their metabolites, the remaining 37 metabolites were further analyzed, with 32 being upregulated and 5 being downregulated (Fig 5B and S4 Table).

**Fig 6:**
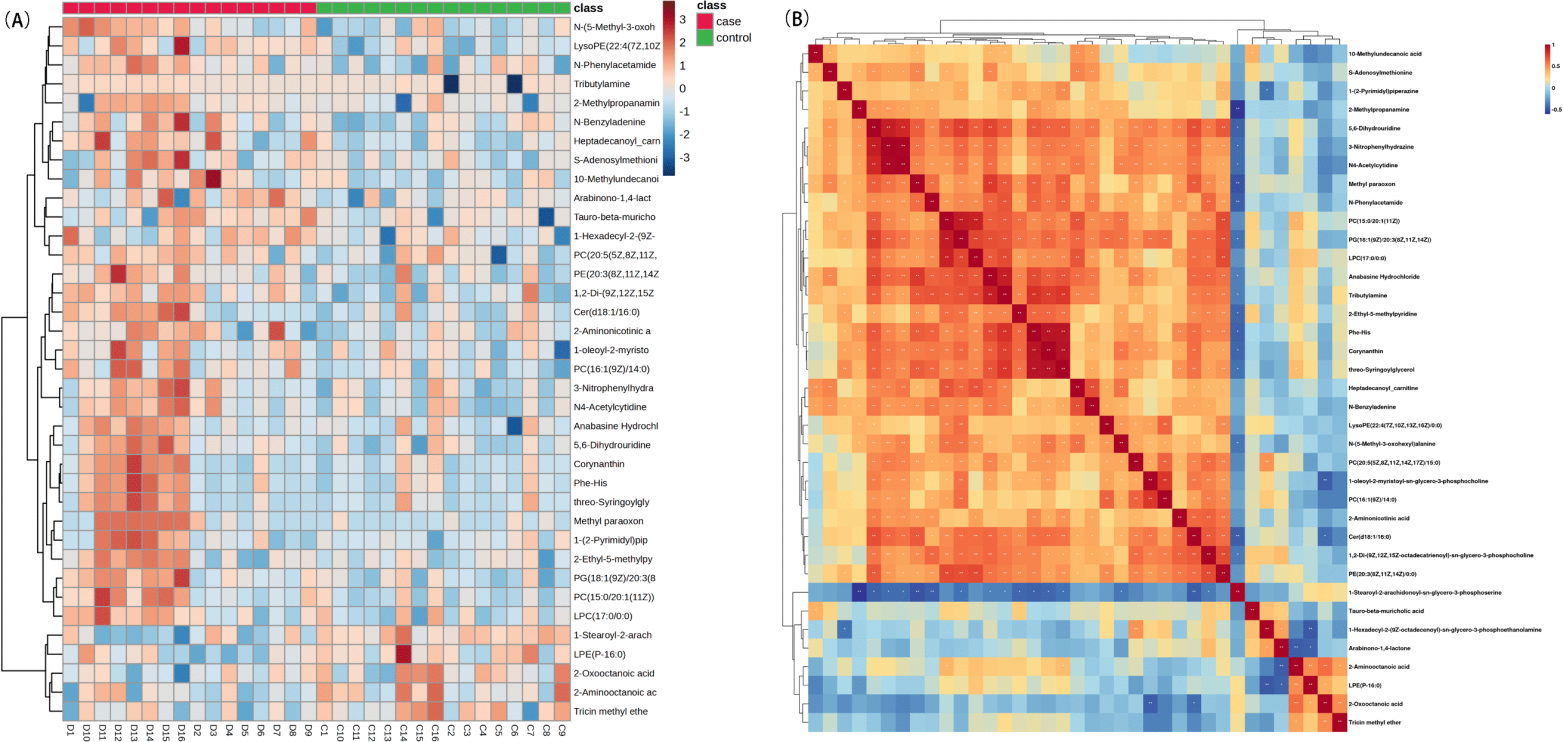
Correlation heatmaps of DEMs in serum of Daur vs. Han patients with CIS. **(A)** Clustered heatmap of DEMs. **(B)** Clustered heatmap of Spearman correlation coefficients.

Cluster analysis. The clustering heatmap revealed the ability of 37 DEMs to distinguish between Daur and Han patients with CIS (Fig 6A). Spearman correlation analysis revealed varying degrees of correlation among the expression levels of these 37 DEMs (Fig 6B).

**Fig 7:**
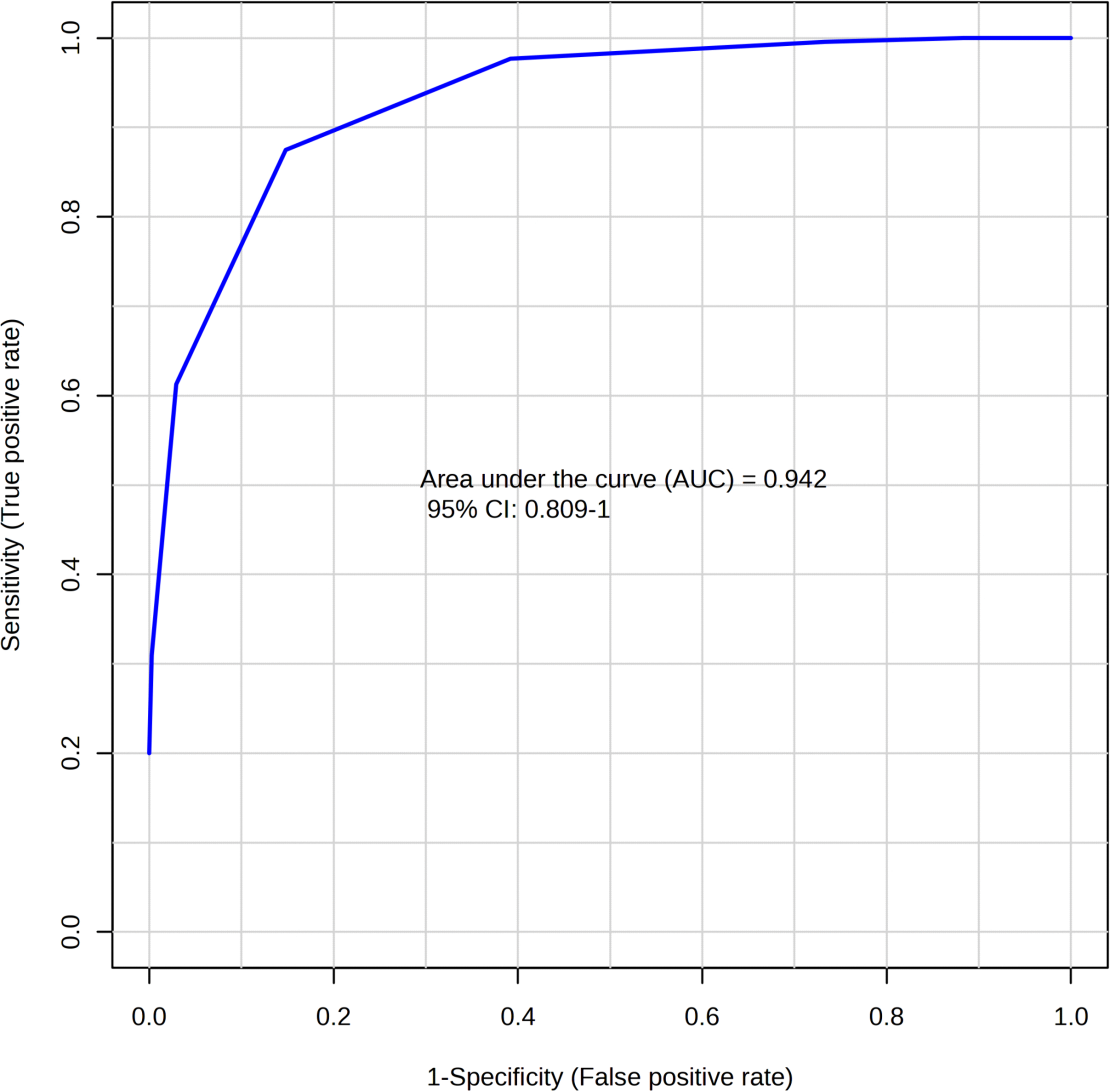
ROC curves of serum DEMs for distinguishing Daur and Han patients with CIS.

ROC curve analysis. Multiple exploratory ROC curves were used to assess the sensitivity and specificity of various metabolites to distinguish between Daur and Han patients with CIS. A multivariate algorithm was established using the metabolite classification method, which is based on PLS-DA, and the feature ranking method, which is built into PLS-DA. The area under the ROC curve (AUC) and 95% CI for the potential DEMs between the Daur and Han patients with CIS were 0.942 (0.809–1.000), with an accuracy of 87.4% (Fig 7). Our initial, exploratory analysis indicated that variations in the selected DEMs showed high sensitivity and specificity, suggesting their preliminary diagnostic potential for distinguishing CIS patients of Daur and Han.

**Fig 8:**
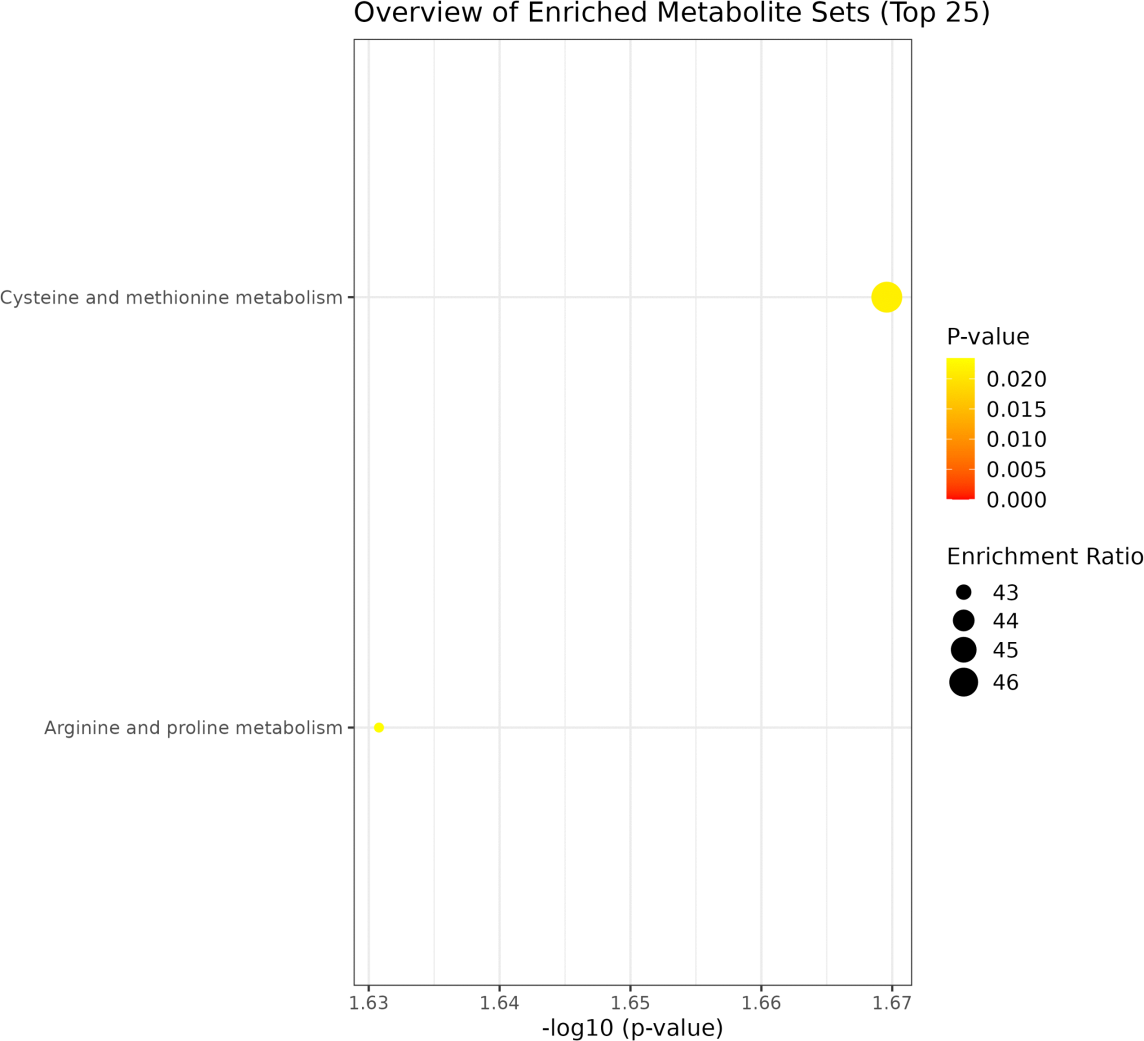
Enrichment analysis of potential DEMs in Daur and Han patients with CIS via KEGG.

Pathway enrichment analysis. The 37 DEMs were introduced into the KEGG database for metabolic pathway analysis, which revealed significant differences in the cysteine and methionine metabolism, arginine and proline metabolism pathways (*P* = 0.021 and *P* = 0.023, respectively) between Daur and Han patients with CIS. These pathways were significantly enriched in CIS due to oxidative stress and vascular dysfunction, but the focus of each pathway differed (Fig 8).

## Discussion

Patients with CIS frequently experience severe outcomes, including mortality and disability, primarily due to disease recurrence. Owing to differences of the onset time, ethnicity, age and other characteristics of the enrolled patients with IS, previous studies have reported significant differences in the upregulation and downregulation patterns of metabolites [20]. Increasing evidence indicates that cold-induced conversion of white adipose tissue (WAT) to brown adipose tissue (BAT) or activation of BAT can also contribute to an increase in energy expenditure [21–23]. Our study focused on patients with CIS in extremely cold rural areas. Selecting patients in the chronic phase helps identify stable metabolic profiles associated with neurological functional deficits and long-term repair mechanisms, reducing confounding effects from acute-phase treatment interventions and dynamic pathophysiological changes. By using nontargeted metabolomics data, the potential DEMs of patients with CIS were identified. The ROC curve of the serum potential DEMs of patients with CIS was constructed. Pathway enrichment analysis revealed significant differences in the galactose metabolic pathway and cysteine and methionine metabolism pathway, revealing the possible mechanisms of energy compensation in the chronic phase of IS. Notably, the serum potential DEMs and ROC curves of Daur patients with CIS were verified, as were the significant differences in the metabolic pathways of cysteine, methionine, arginine and proline. On the basis of differential expression analysis and OPLS-DA, among 29 potential DEMs identified in residents of the extremely cold region of Qiqihar who developed CIS, four metabolites, S-adenosylmethionine, cystine, D-galactose, and normetanephrine, were identified that have been consistently recognized as biomarkers of CIS in multiple studies. S-Adenosylmethionine is an essential metabolite for transmethylation reactions and a substrate for polyamine synthesis [24]. Its metabolic dysregulation is frequently observed in CIS patients and is linked to the homocysteine metabolic pathway [25]. Accumulating evidence suggests its role in poststroke blood‒brain barrier disruption and neuronal repair [26,27]. Cystine, an oxidative stress marker and component of the cystine/glutamate antiporter, is significantly elevated during ischemia reperfusion injury [28], which is correlated with free radical-induced cardiovascular damage after stroke [29]. D-Galactose, a metabolite associated with chronic inflammation and aging, may exacerbate postischemic neural injury through advanced glycation end products (AGEs) [30]. Abnormal serum levels of AGEs have been documented in stroke patients [31]. Normetanephrine, a catecholamine metabolite reflecting sympathetic activity, is implicated in poststroke stress responses and blood pressure fluctuations [32,33]. Clinical studies have demonstrated its association with stroke severity [34].

Our study confirmed that, through ROC analysis, these metabolites showed statistical significance in distinguishing CIS from healthy control groups, but their AUC values were moderate, insufficient to support their use as independent diagnostic biomarkers. In contrast, existing studies typically achieve higher diagnostic efficacy based on broader metabolomic analyses or combinations of multiple biomarkers. For example, Tiedt S et al have confirmed extremely high discriminatory ability with 30 metabolites (AUC = 0.961) [35]. Notably, metabolomic features may reflect early systemic metabolic disturbances that imaging or cognitive tests have not yet captured, and they offer better non-invasiveness and accessibility. Additionally, this study found that changes in metabolites are consistent with key pathophysiological processes reported in previous study, such as atherosclerosis [36], neurological function recovery [37], and amino acid metabolic disorders [38]. Specifically, S-adenosylmethionine and cystine in this study are related to atherosclerosis and amino acid metabolic disorders, while normetanephrine and ectoine are associated with neurological function recovery. Therefore, although the metabolite combination identified in the current study lacks independent diagnostic value, it provides preliminary candidate biomarkers and mechanistic hypotheses for subsequent prospective cohort studies.

Our study revealed that among the 29 potential DEMs associated with CIS, ectoine and N-methyl-L-proline (hygric acid) have not been reported previously. To the best of our knowledge, changes in ectoine levels have not been reported in the context of human ischemic stroke before. It may serve as a new potential metabolite involved in CIS. However, ectoine has been associated with mitochondrial dysfunction in Alzheimer’s disease [39]. Although N-Methyl-L-proline has been linked to colon polyps [40], its role in CIS remains poorly understood. Our research findings suggest that it may be a metabolite worthy of attention during the post-stroke recovery process. Two metabolites were newly discovered in this study, including PC (18:3(9Z,12Z,15Z)/18:3(9Z,12Z,15Z)), which is a polyunsaturated phosphatidylcholine involved in the sphingolipid metabolism pathway [41]. It is related to a poptosis and membrane stability and may serve as a new target for stroke treatment [42]. 22-Acetylpriverogenin B is a steroidal saponin derivative that may regulate neuroinflammation and tissue damage through microglial activation or polarization, potentially having functional benefits in the treatment of ischemic stroke [43]. Ganoderiol C and cis-resveratrol 4’-sulfate have neuroprotective potential but lack specific evidence for stroke involvement [44,45]. 5-Feruloylquinic acid, a bioactive compound with anti-inflammatory and antioxidant properties, is associated with vascular inflammatory diseases but its relationship with stroke mechanisms remains unclear [46]. These three metabolites require further validation. This study is expected to provide auxiliary support for clinical diagnosis by identifying metabolomic features of CIS patients. The potential DEMs discovered can not only serve as an effective supplement to existing clinical and imaging evaluations to identify patients in the recovery phase, but also provide reference for subsequent adjustments of individualized treatment strategies.

Further KEGG pathway analysis identified galactose metabolism, as well as cysteine and methionine metabolism as a critical pathway in CIS patients. IS is significantly associated with the galactose metabolism signaling pathway, which is associated with energy imbalance and cognitive impairment caused by cerebral ischemia [47,48]. Galactose can combine with ceramide to synthesize galactocerebrosides, a type of sphingolipid [49]. When this metabolic pathway is differentially regulated in patients with chronic stroke, it may indicate that their demand for related metabolic substrates has changed. Excess galactose accumulation leads to the production of free radicals, which leads to oxidative stress and inflammation [50]. Lactose in dairy products is considered the main source of galactose. The results of the dietary frequency questionnaire in this study showed that there was no statistically significant difference in dairy product intake between CIS patients and healthy controls (Z = −1.246, *P* = 0.213). Therefore, the inter-group differences in the galactose metabolism pathway are more likely to result from metabolic disturbances caused by the CIS disease state itself, rather than confounding factors from dietary intake. This study selected patients from the Daur and Han ethnic groups for comparison, as previous epidemiological data indicated that the prevalence of hypertension among the Daur population residing in extremely cold regions was significantly higher than the national average [51]. The recurrence rates of stroke patients in China are 3.6% at three months and 5.6% at twelve months [52]. Hypertension is significantly associated with recurrent stroke [53]. The multivariate Logistic regression analysis of our study of stroke patients revealed that a history of hypertension was a risk factor for CIS. The Daur is one of 55 ethnic minorities in China and they have lived in the northern part of the country for 300 years. The common edible plants used by the Daur, such as those from rosaceae and asteraceae, are closely related to traditional Chinese medicine and the treatment of diseases [54]. The unique metabolic characteristics of the Daur ethnic group may be closely related to their traditional culture, dietary patterns and other ethnic-specific factors. This study initially revealed the potential association between ethnic background and metabolic profiles by analyzing the differential metabolites between Daur and Han Chinese patients with CIS. Among the 37 potential DEMs identified in the serum of Daur patients with CIS in this study. A total of 172 alleles at 19 X-STR loci were observed in the Daur population, revealing the phylogenetic similarities and differences between the Daur and other ethnic minorities in China [55]. The genetic susceptibility to hypertension and stroke in the Daur population may be related to variations in the alpha-1A adrenergic receptor (ADRA1A) gene. ADRA1A can regulate changes in vascular tension through sympathetic nerve contraction [56], which is closely related to hypertension [57,58]. Although the current analysis did not fully explain the mechanism underlying the differences in metabolic products of stroke among different ethnic groups, as the study focused on the metabolic characteristics of CIS in the Daur population in extremely cold regions, its results provide preliminary clues for subsequent precise interventions targeting the health of ethnic minorities. However, the exact causal relationship between them still needs to be further verified by large-scale prospective cohort studies. Further KEGG enrichment analysis revealed that cysteine and methionine metabolism, and arginine and proline metabolism pathways are key pathways affecting Daur patients with CIS. Previous studies have linked methionine and cysteine metabolic disorders to stroke onset. When the concentrations of homocysteine and cysteine are increased in blood plasma, it causes hyperhomocysteinemia, leading to the development of atherosclerosis and thrombotic complications. This process can be associated with elevated levels of reactive oxygen species and increased expression of proinflammatory cytokines [59]. Arginine and proline biosynthesis may be involved in the development of CIS by influencing biological processes such as energy failure, oxidative stress, and apoptosis [60]. However, after ischemia, when glucose oxidative metabolism in the brain decreases, an increase in the metabolic conversion of these amino acids into alternative substrates is observed [61]. It seems that cells with an oxygen restriction might prefer or reuse molecules that work as alternative energy substrates [62]. This further exacerbates metabolic disorders in stroke patients. Metabolic disorders of cholesterol in stroke patients have been confirmed in recent studies [63]. This study analyzed potential DEMs in the serum of CIS patients and revealed significant differences in pathways related to galactose metabolism, cysteine and methionine metabolism, and arginine and proline metabolism relative to healthy controls.

Our study has several limitations. The primary limitation is the modest sample size, which reduces statistical power, limits generalizability, and increases the risk of false discoveries, particularly in subgroup analyses. Additionally, the strict inclusion criteria and the small population base in these remote, extremely cold regions further constrained recruitment. Therefore, we explicitly present this work as an exploratory study. Its findings are preliminary and require validation in larger cohorts. Despite these constraints, as with similar exploratory metabolomics studies [64], our analysis provides valuable preliminary data and identifies specific metabolic targets for future large-scale validation in this unique population.

Additionally, this study identified new candidate metabolites of CIS patients through nontargeted metabolomics, such as PC (18:3(9Z,12Z,15Z)/18:3(9Z,12Z,15Z)) and 22-Acetylpriverogenin B. However, the sensitivity and specificity of these markers are still unclear because of the lack of independent cohort validation. Although the findings of this study partially align with the known galactose metabolic pathways of CIS [65], the clinical translation of these findings still requires further verification through prospective studies.

Furthermore, this study focused on elderly CIS patients residing in extremely cold regions, and their metabolic characteristics may be affected by multiple factors such as comorbidities of hypertension or diabetes, polypharmacy, and age-related physiological decline. Due to the retrospective design of this study, we were unable to systematically collect detailed medication information from patients, including drug types, dosages, and treatment durations, which constitutes the main limitation of this study. Although we excluded some confounding factors through questionnaires and matching, the broad characteristics of nontargeted metabolomics may still introduce some confounding factors. Hypertension can lead to vascular endothelial dysfunction, enhanced oxidative stress, and a chronic inflammatory state [66–68]. This may affect amino acid metabolism, glucose metabolism, oxidative stress, lipid profile, steroid hormones, and glucose metabolic pathways [69]. Drug therapy is an important influencing factor in the metabolic profile of stroke patients. For example, the effect of statins not only lowers low-density lipoprotein cholesterol but can also cause significant changes in the lipid profile of statin users [70]. D-galactose and cystine may be associated with diabetes, hypertension, and stroke simultaneously, and there is a lack of specificity for determining the specific metabolic products of stroke [71,72].

Future studies should overcome this limitation through systematic design. Ideally, this would include prospectively and standardly collecting detailed medication data at both baseline and follow-up stages. Where ethically and clinically feasible, enrolling a subgroup of non-treatment newly diagnosed patients, or establishing a standardized short-term medication washout period prior to sampling. Simultaneously, employing more advanced statistical methods or recruiting multicenter cohorts with more diverse medication patterns. These strategies will help to better distinguish stroke-specific metabolic alterations from the confounding effects of pharmacotherapy.

Finally, this study used univariate statistics to screen differentially abundant metabolites, which can initially identify significant targets, but fail to analyze the synergistic effects or nonlinear associations among metabolic pathways. This limitation may lead to the omission of some potential biomarkers. Recent studies have shown that obtaining gene expression data related to CIS from comprehensive databases and using machine learning algorithms to select candidate genes is beneficial [73]. Current research findings are to be used as a supplement to, rather than a replacement for, clinical standards. In future studies, the use of multivariate modeling, network analysis, and multiomics integration will facilitate the clinical identification of markers to predict the risk of recurrence in patients with CIS and provide guidance for targeted intervention.

In conclusion, we identified potential metabolites related to CIS through metabolomics. The results of this study indicate that serum metabolomics analysis has significant potential for identifying biomarkers to predict the risk of recurrent CIS. In the future, it is necessary to evaluate these biomarkers in stroke patients further to better understand their predictive capabilities and whether they can be used to determine patients’ disease recovery status. This study lays the foundation for developing valuable diagnostic tools and exploring new therapeutic targets, aiming to provide evidence to support rehabilitation treatment and treatment decisions for patients with CIS.

## Supporting information

S1 Fig The correlation analysis of QC samples. (TIF)

S1 Table Specific confidence levels of metabolites. (PDF)

S2 Table Internal standard response stability in QC samples. (PDF)

S3 Table The potential DEMs between ischemic stroke patients and healthy controls. (PDF)

S4 Table The potential DEMs between Daur and Han patients with CIS. (PDF)

## References

[1] Shichita T, Ooboshi H, Yoshimura A. Neuroimmune mechanisms and therapies mediating post-ischaemic brain injury and repair. Nat Rev Neurosci. 2023;24(5):299–312. doi: 10.1038/s41583-023-00690-0 36973481

[2] Sifat AE, Nozohouri S, Archie SR, Chowdhury EA, Abbruscato TJ. Brain Energy metabolism in ischemic stroke: Effects of smoking and diabetes. Int J Mol Sci. 2022;23(15):8512. doi: 10.3390/ijms23158512 35955647 PMC9369264

[3] Chen Z, Liu P, Xia X, Wang L, Li X. The underlying mechanisms of cold exposure-induced ischemic stroke. Sci Total Environ. 2022;834:155514. doi: 10.1016/j.scitotenv.2022.155514 35472344

[4] Maushart CI, Senn JR, Loeliger RC, Siegenthaler J, Bur F, Fischer JGW, et al. Resting energy expenditure and cold-induced thermogenesis in patients with overt hyperthyroidism. J Clin Endocrinol Metab. 2022;107(2):450–61. 34570185 10.1210/clinem/dgab706 PMC8764338

[5] Compher C, Frankenfield D, Keim N, Roth-Yousey L, Evidence analysis working group. Best practice methods to apply to measurement of resting metabolic rate in adults: a systematic review. J Am Diet Assoc. 2006;106(6):881–903. 16720129 10.1016/j.jada.2006.02.009

[6] Tu WJ, Zhao Z, Yin P, Cao L, Zeng J, Chen H, et al. Estimated burden of stroke in China in 2020. JAMA Netw Open. 2023;6(3):e231455. 10.1001/jamanetworkopen.2023.1455 PMC9982699 36862407

[7] National Health Commission of the People’s Republic of China. China health statistics yearbook. Beijing: Peking Union Medical College Press. 2021.

[8] Herpich F, Rincon F. Management of acute ischemic stroke. Crit Care Med. 2020;48(11):1654–63. 32947473 10.1097/CCM.0000000000004597 PMC7540624

[9] Fuller PJ, Yao Y-Z, Yang J, Young MJ. Structural determinants of activation of the mineralocorticoid receptor: an evolutionary perspective. J Hum Hypertens. 2021;35(2):110–6. doi: 10.1038/s41371-020-0360-2 32467588

[10] Bernardo-Castro S, Sousa JA, Brás A, Cecília C, Rodrigues B, Almendra L, et al. Pathophysiology of blood-brain barrier permeability throughout the different stages of ischemic stroke and its implication on hemorrhagic transformation and recovery. Front Neurol. 2020;11:594672. doi: 10.3389/fneur.2020.594672 33362697 PMC7756029

[11] Albers GW, Marks MP, Kemp S, Christensen S, Tsai JP, Ortega-Gutierrez S, et al. Thrombectomy for stroke at 6 to 16 hours with selection by perfusion imaging. N Engl J Med. 2018;378(8):708–18. doi: 10.1056/NEJMoa1713973 29364767 PMC6590673

[12] Nogueira RG, Jadhav AP, Haussen DC, Bonafe A, Budzik RF, Bhuva P, et al. Thrombectomy 6 to 24 hours after stroke with a mismatch between deficit and infarct. N Engl J Med. 2018;378(1):11–21. 29129157 10.1056/NEJMoa1706442

[13] Varona JF. Long-term prognosis of ischemic stroke in young adults. Stroke Res Treat. 2010;2011:879817. 21197408 10.4061/2011/879817 PMC3010699

[14] Yang L, Wang Y, Cai H, Wang S, Shen Y, Ke C. Application of metabolomics in the diagnosis of breast cancer: a systematic review. J Cancer. 2020;11(9):2540–51. 32201524 10.7150/jca.37604 PMC7066003

[15] Donatti A, Canto AM, Godoi AB, da Rosa DC, Lopes-Cendes I. Circulating metabolites as potential biomarkers for neurological disorders-metabolites in neurological disorders. Metabolites. 2020;10(10):389. doi: 10.3390/metabo10100389 33003305 PMC7601919

[16] Zahoor I, Rui B, Khan J, Datta I, Giri S. An emerging potential of metabolomics in multiple sclerosis: A comprehensive overview. Cell Mol Life Sci. 2021;78(7):3181–203. 33449145 10.1007/s00018-020-03733-2 PMC8038957

[17] Au A. Metabolomics and lipidomics of ischemic stroke. Adv Clin Chem. 2018;85:31–69. doi: 10.1016/bs.acc.2018.02.002 29655461

[18] Montaner J, Ramiro L, Simats A, Tiedt S, Makris K, Jickling GC, et al. Multilevel omics for the discovery of biomarkers and therapeutic targets for stroke. Nat Rev Neurol. 2020;16(5):247–64. doi: 10.1038/s41582-020-0350-6 32322099

[19] Zhang R, Meng J, Wang X, Pu L, Zhao T, Huang Y, et al. Metabolomics of ischemic stroke: Insights into risk prediction and mechanisms. Metab Brain Dis. 2022;37(7):2163–80. doi: 10.1007/s11011-022-01011-7 35612695

[20] Shin TH, Lee DY, Basith S, Manavalan B, Paik MJ, Rybinnik I, et al. Metabolome changes in cerebral ischemia. Cells. 2020;9(7):1630. doi: 10.3390/cells9071630 32645907 PMC7407387

[21] Palmer BF, Clegg DJ. Non-shivering thermogenesis as a mechanism to facilitate sustainable weight loss. Obes Rev. 2017;18(8):819–31. doi: 10.1111/obr.12563 28547916

[22] Lee P, Greenfield JR. Non-pharmacological and pharmacological strategies of brown adipose tissue recruitment in humans. Mol Cell Endocrinol. 2015;418 Pt 2:184–90. doi: 10.1016/j.mce.2015.05.025 26026310

[23] Eyolfson DA, Tikuisis P, Xu X, Weseen G, Giesbrecht GG. Measurement and prediction of peak shivering intensity in humans. Eur J Appl Physiol. 2001;84(1–2):100–6. doi: 10.1007/s004210000329 11394237

[24] Quinn C, Rico MC, Merali C, Merali S. Dysregulation of S-adenosylmethionine Metabolism in Nonalcoholic Steatohepatitis Leads to Polyamine Flux and Oxidative Stress. Int J Mol Sci. 2022;23(4):1986. doi: 10.3390/ijms23041986 35216100 PMC8878801

[25] Wan C, Zong RY, Chen XS. The new mechanism of cognitive decline induced by hypertension: High homocysteine-mediated aberrant DNA methylation. Front Cardiovasc Med. 2022;9:928701. 36352848 10.3389/fcvm.2022.928701 PMC9637555

[26] Liu A, Zhu XJ, Sun WD, Bi SZ, Zhang CY, Lai SY, et al. Nicotinamide N-methyltransferase as a potential therapeutic target for neurodegenerative disorders: Mechanisms, challenges, and future directions. Exp Neurol. 2025;389:115253. 40221009 10.1016/j.expneurol.2025.115253

[27] Kang W, Zhang Y, Cui W, Meng H, Zhang D. Folic Acid promotes peripheral nerve injury repair via regulating DNM3-AKT pathway through mediating methionine cycle metabolism. Neuromolecular Med. 2025;27(1):23. doi: 10.1007/s12017-025-08845-1 40163256 PMC11958391

[28] Hu M, Huang J, Chen L, Sun X-R, Yao Z-M, Tong X-H, et al. Upregulation of CDGSH iron sulfur domain 2 attenuates cerebral ischemia/reperfusion injury. Neural Regen Res. 2023;18(7):1512–20. doi: 10.4103/1673-5374.355766 36571356 PMC10075131

[29] Maity J, Dey T, Banerjee A, Chattopadhyay A, Das AR, Bandyopadhyay D. Melatonin ameliorates myocardial infarction in obese diabetic individuals: The possible involvement of macrophage apoptotic factors. J Pineal Res. 2023;74(2):e12847. doi: 10.1111/jpi.12847 36456538

[30] Haase S, Kuhbandner K, Mühleck F, Gisevius B, Freudenstein D, Hirschberg S, et al. Dietary galactose exacerbates autoimmune neuroinflammation via advanced glycation end product-mediated neurodegeneration. Front Immunol. 2024;15:1367819. doi: 10.3389/fimmu.2024.1367819 39185426 PMC11341352

[31] Chen J, Lv Y-N, Li X-B, Xiong J-J, Liang H-T, Xie L, et al. Urinary metabolite signatures for predicting elderly stroke survivors with depression. Neuropsychiatr Dis Treat. 2021;17:925–33. doi: 10.2147/NDT.S299835 33790561 PMC8007561

[32] Golani T, Fishman B, Sharabi Y, Olswang-Kutz Y, Leibowitz A, Grossman E, et al. The association between systolic blood pressure reduction during clonidine suppression testing and the decrease in plasma catecholamines and metanephrines. J Clin Hypertens (Greenwich). 2020;22(10):1924–31. doi: 10.1111/jch.14014 32882089 PMC8029920

[33] Tella SH, Jha A, Taïeb D, Horvath KA, Pacak K. Comprehensive review of evaluation and management of cardiac paragangliomas. Heart. 2020;106(16):1202–10. doi: 10.1136/heartjnl-2020-316540 32444502 PMC7482425

[34] Madhok J, Kloosterboer A, Venkatasubramanian C, Mihm FG. Catecholamine-induced cerebral vasospasm and multifocal infarctions in pheochromocytoma. Endocrinol Diabetes Metab Case Rep. 2020;2020:20–0078. doi: 10.1530/EDM-20-0078 32820130 PMC7487175

[35] Tiedt S, Brandmaier S, Kollmeier H, Duering M, Artati A, Adamski J, et al. Circulating metabolites differentiate acute ischemic stroke from stroke mimics. Ann Neurol. 2020;88(4):736–46. doi: 10.1002/ana.25859 32748431

[36] Vorkas PA, Shalhoub J, Lewis MR, Spagou K, Want EJ, Nicholson JK, et al. Metabolic phenotypes of carotid atherosclerotic plaques relate to stroke risk: An exploratory study. Eur J Vasc Endovasc Surg. 2016;52(1):5–10. doi: 10.1016/j.ejvs.2016.01.022 27231199

[37] Suissa L, Guigonis J-M, Graslin F, Doche E, Osman O, Chau Y, et al. Metabolome of cerebral thrombi reveals an association between high glycemia at stroke onset and good clinical outcome. Metabolites. 2020;10(12):483. doi: 10.3390/metabo10120483 33255770 PMC7760729

[38] Chumachenko MS, Waseem TV, Fedorovich SV. Metabolomics and metabolites in ischemic stroke. Rev Neurosci. 2021;33(2):181–205. doi: 10.1515/revneuro-2021-0048 34213842

[39] Xingrong L, Gorish BMT, Qaria MA, Hussain A, Abdelmula WIY, Zhu D. Unlocking Ectoine’s postbiotic therapeutic promise: mechanisms, applications, and future directions. Probiotics Antimicrob Proteins. 2025;:10.1007/s12602-025-10506–5. doi: 10.1007/s12602-025-10506-5 40072821

[40] Xie Y, Jin Y, Liu Z, Li J, Tao Q, Wu Y, et al. Identification of diagnostic biomarkers for colorectal polyps based on noninvasive urinary metabolite screening and construction of a nomogram. Cancer Med. 2025;14(7):e70762. doi: 10.1002/cam4.70762 40200572 PMC11978731

[41] Zhang D, Yuan C, An X, Guo T, Lu Z, Liu J. Transcriptome and metabolome revealed the effects of hypoxic environment on ovarian development of Tibetan sheep. Genomics. 2025;117(1):110973. doi: 10.1016/j.ygeno.2024.110973 39631551

[42] Sherratt SCR, Juliano RA, Mason RP. Eicosapentaenoic acid (EPA) has optimal chain length and degree of unsaturation to inhibit oxidation of small dense LDL and membrane cholesterol domains as compared to related fatty acids in vitro. Biochim Biophys Acta Biomembr. 2020;1862(7):183254. doi: 10.1016/j.bbamem.2020.183254 32135144

[43] Kang C, Sang Q, Liu D, Wang L, Li J, Liu X. Polyphyllin I alleviates neuroinflammation after cerebral ischemia-reperfusion injury via facilitating autophagy-mediated M2 microglial polarization. Mol Med. 2024;30(1):59. 38745316 10.1186/s10020-024-00828-5 PMC11094947

[44] Liu Y, Feng X, Wang J, Li M. Neuroprotective effect of ganoderic acid against focal ischemic stroke induced by middle cerebral artery occlusion in the rats via suppression of oxidative stress and inflammation. Dokl Biochem Biophys. 2024;518(1):361–71. doi: 10.1134/S1607672924600313 39023671

[45] Wang Q, Yu Q, Wu M. Antioxidant and neuroprotective actions of resveratrol in cerebrovascular diseases. Front Pharmacol. 2022;13:948889. doi: 10.3389/fphar.2022.948889 36133823 PMC9483202

[46] Smeriglio A, Denaro M, D’Angelo V, Germanò MP, Trombetta D. Antioxidant, anti-inflammatory and anti-angiogenic properties of citrus lumia juice. Front Pharmacol. 2020;11:593506. doi: 10.3389/fphar.2020.593506 33343362 PMC7744484

[47] Lu R, Yang J, Fan R, Li H, Liu Y, Wang T, et al. Prophylactic administration of Xuesaitong soft capsule ameliorating depression-like behaviors in rats after ischemic stroke by modulating metabolic disturbance. Phytomedicine. 2025;145:156997. doi: 10.1016/j.phymed.2025.156997 40570557

[48] Wu H-T, Yu Y, Li X-X, Lang X-Y, Gu R-Z, Fan S-R, et al. Edaravone attenuates H2O2 or glutamate-induced toxicity in hippocampal neurons and improves AlCl3/D-galactose induced cognitive impairment in mice. Neurotoxicology. 2021;85:68–78. doi: 10.1016/j.neuro.2021.05.005 34004234

[49] Gaspari E, Koehorst JJ, Frey J, Martins Dos Santos VAP, Suarez-Diez M. Galactocerebroside biosynthesis pathways of Mycoplasma species: An antigen triggering Guillain-Barré-Stohl syndrome. Microb Biotechnol. 2021;14(3):1201–11. doi: 10.1111/1751-7915.13794 33773097 PMC8085918

[50] Zhang Y, Ma R, Deng Q, Wang W, Cao C, Yu C, et al. S-adenosylmethionine improves cognitive impairment in D-galactose-induced brain aging by inhibiting oxidative stress and neuroinflammation. J Chem Neuroanat. 2023;128:102232. doi: 10.1016/j.jchemneu.2023.102232 36632907

[51] Wang L, Fu L, Han Y, Qian X. Prevalence and risk factors of hypertension in rural Daur population. Chin J Public Health. 2005;21(7):814–5.

[52] Tu WJ, Wang LD, Special writing group of China stroke surveillance report. China stroke surveillance report 2021. Mil Med Res. 2023;10(1):33. Kwok GYR, Chen RWR, 37468952 10.1186/s40779-023-00463-x PMC10355019

[53] Leow TA, Kok C, Yeong N, Teo YH, et al. Recurrent ischemic stroke in young adults: A multicenter cohort study, systematic review, and meta-analysis. Int J Stroke. 2026;21(1):24–35. 40292815 10.1177/17474930251340799

[54] Bi Y, Gao F, Guo J, Yao X, Wang A, Liu H, et al. An ethnobotanical survey on the medicinal and edible plants used by the Daur people in China. J Ethnobiol Ethnomed. 2024;20(1):55. doi: 10.1186/s13002-024-00695-8 38790060 PMC11127305

[55] Liu Y, Ye Y, Yi X-Q, Zhang J-H, Fan G-Y, Hao D-Y. Phylogenetic analyses of the 19 STR loci in X chromosome revealed discriminant forensic characteristics for Chinese Daur and Oroqen minorities. Int J Legal Med. 2022;136(2):551–3. doi: 10.1007/s00414-021-02697-7 34510269

[56] Baranova T, Podyacheva E, Zemlyanukhina T, Berlov D, Danilova M, Glotov O, et al. Vascular reactions of the diving reflex in men and women carrying different ADRA1A Genotypes. Int J Mol Sci. 2022;23(16):9433. doi: 10.3390/ijms23169433 36012699 PMC9409260

[57] Kim J-D, Kwon C, Nakamura K, Muromachi N, Mori H, Muroi S-I, et al. Increased angiotensin II coupled with decreased Adra1a expression enhances cardiac hypertrophy in pregnancy-associated hypertensive mice. J Biol Chem. 2023;299(3):102964. doi: 10.1016/j.jbc.2023.102964 36736425 PMC10011504

[58] Kang J-O, Ha TW, Jung H-U, Lim JE, Oh B. A cardiac-null mutation of Prdm16 causes hypotension in mice with cardiac hypertrophy via increased nitric oxide synthase 1. PLoS One. 2022;17(7):e0267938. doi: 10.1371/journal.pone.0267938 35862303 PMC9302805

[59] Gospodarczyk A, Marczewski K, Gospodarczyk N, Widuch M, Tkocz M, Zalejska-Fiolka J. Homocysteine and cardiovascular disease - a current review. Wiad Lek. 2022;75(11 pt 2):2862–6. 36591781 10.36740/WLek202211224

[60] Jia J, Zhang H, Liang X, Dai Y, Liu L, Tan K, et al. Application of metabolomics to the discovery of biomarkers for ischemic stroke in the murine model: A comparison with the clinical results. Mol Neurobiol. 2021;58(12):6415–26. 34532786 10.1007/s12035-021-02535-2

[61] Karadima E, Chavakis T, Alexaki VI. Arginine metabolism in myeloid cells in health and disease. Semin Immunopathol. 2025;47(1):11. doi: 10.1007/s00281-025-01038-9 39863828 PMC11762783

[62] Pascual JM, Carceller F, Roda JM, Cerdán S. Glutamate, glutamine, and GABA as substrates for the neuronal and glial compartments after focal cerebral ischemia in rats. Stroke. 1998;29(5):1048–56; discussion 1056-7. doi: 10.1161/01.str.29.5.1048 9596256

[63] Kong Y, Feng Y-Q, Lu Y-T, Feng S-S, Huang Z, Wang Q-Y, et al. Predictive serum biomarkers of patients with cerebral infarction. Neurol Res. 2022;44(4):331–41. doi: 10.1080/01616412.2021.1987055 34763612

[64] Li K, Wang K, Xu S-X, Xie X-H, Tang Y, Zhang L, et al. In vivo evidence of increased vascular endothelial growth factor in patients with major depressive disorder. J Affect Disord. 2025;368:151–9. doi: 10.1016/j.jad.2024.09.073 39278472

[65] Li D-H, Su Y-F, Sun C-X, Fan H-F, Gao W-J. A network pharmacology-based identification study on the mechanism of xiao-xu-ming decoction for cerebral ischemic stroke. Evid Based Complement Alternat Med. 2020;2020:2507074. doi: 10.1155/2020/2507074 33133212 PMC7593742

[66] Backston K, Morgan J, Patel S, Koka R, Hu J, Raina R. Oxidative stress and endothelial dysfunction: The pathogenesis of pediatric hypertension. Int J Mol Sci. 2025;26(11):5355. doi: 10.3390/ijms26115355 40508164 PMC12154540

[67] Chaudhary P, Pandey A, Azad CS, Tia N, Singh M, Gambhir IS. Association of oxidative stress and endothelial dysfunction in hypertension. Anal Biochem. 2020;590:113535. doi: 10.1016/j.ab.2019.113535 31821803

[68] Dinh QN, Drummond GR, Sobey CG, Chrissobolis S. Roles of inflammation, oxidative stress, and vascular dysfunction in hypertension. Biomed Res Int. 2014;2014:406960. doi: 10.1155/2014/406960 25136585 PMC4124649

[69] Onuh JO, Qiu H. Metabolic profiling and metabolites fingerprints in human hypertension: Discovery and potential. Metabolites. 2021;11(10):687. doi: 10.3390/metabo11100687 34677402 PMC8539280

[70] Hoshi RA, Alotaibi M, Liu Y, Watrous JD, Ridker PM, Glynn RJ, et al. One-year effects of high-intensity statin on bioactive lipids: findings from the jupiter trial. Arterioscler Thromb Vasc Biol. 2024;44(7):e196–206. doi: 10.1161/ATVBAHA.124.321058 38841856 PMC11209760

[71] Sun C, Wang L, Huang H, Zheng Z, Xu X, Wang H, et al. Mitigation of gestational diabetes-induced endothelial dysfunction through FGF21-NRF2 pathway activation involving L-Cystine. Biochim Biophys Acta Mol Basis Dis. 2024;1870(7):167329. doi: 10.1016/j.bbadis.2024.167329 38960053

[72] Yang S, Wang Z, Guo M, Du M, Wen X, Geng L, et al. UPLC-MS-based serum metabolomics reveals potential biomarkers of ang ii-induced hypertension in mice. Front Cardiovasc Med. 2021;8:683859. doi: 10.3389/fcvm.2021.683859 34026879 PMC8131677

[73] Li T, Li H, Zhang S, Wang Y, He J, Kang J. Transcriptome sequencing-based screening of key melatonin-related genes in ischemic stroke. Int J Mol Sci. 2024;25(21):11620. doi: 10.3390/ijms252111620 39519172 PMC11547107

