# Supplementary figures and images for: Nontargeted metabolomics analysis of potential biomarkers for patients with chronic ischemic stroke in extremely cold rural regions: An exploratory case-control study
Source: PLoS One. 2026 Feb 20;21(2):e0341966. doi: 10.1371/journal.pone.0341966 (PMC12923066; doi:10.1371/journal.pone.0341966)

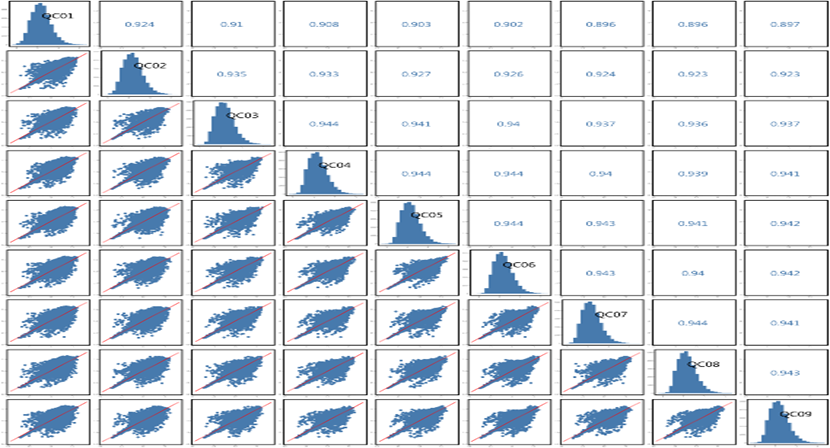

Supplement: S1 Fig — (TIF) [file pone.0341966.s001.tif]
