# Supplementary material for: Nontargeted metabolomics analysis of potential biomarkers for patients with chronic ischemic stroke in extremely cold rural regions: An exploratory case-control study
Source: PLoS One. 2026 Feb 20;21(2):e0341966. doi: 10.1371/journal.pone.0341966 (PMC12923066; doi:10.1371/journal.pone.0341966)
Supplement: S1 Table — (PDF) [file pone.0341966.s002.pdf]

S1 Table. Specific confidence levels of metabolites

Confidence level	Core meaning	Key evidence	Number of studies
Level 1	Identified	Comparison with reference standards (retention time, MS/MS fragment spectrum matching)	434
Level 2	Commented	Accurate mass-to-charge ratio, matching of database MS/MS spectra	809
Level 3	Commented	Comparison with predicted retention time, and MS/MS fragment spectrum	65
Level 4	Unknown	Retain time	18639
