# Supplementary material for: Nontargeted metabolomics analysis of potential biomarkers for patients with chronic ischemic stroke in extremely cold rural regions: An exploratory case-control study
Source: PLoS One. 2026 Feb 20;21(2):e0341966. doi: 10.1371/journal.pone.0341966 (PMC12923066; doi:10.1371/journal.pone.0341966)
Supplement: S2 Table — (PDF) [file pone.0341966.s003.pdf]

S2 Table. Internal standard response stability in QC samples

Internal standard	rt	mz	rsd
IS1	119.7	92.03	0.03
IS2	158	133.10	0.04
IS3	102.5	183.08	0.03
IS4	194.4	85.13	0.02
IS5	161.9	127.14	0.03
IS6	226.6	154.07	0.11
