# Supplementary material for: Nontargeted metabolomics analysis of potential biomarkers for patients with chronic ischemic stroke in extremely cold rural regions: An exploratory case-control study
Source: PLoS One. 2026 Feb 20;21(2):e0341966. doi: 10.1371/journal.pone.0341966 (PMC12923066; doi:10.1371/journal.pone.0341966)
Supplement: S3 Table — (PDF) [file pone.0341966.s004.pdf]

S4 Table. The potential DEMs between ischemic stroke patients and healthy controls

MS_name	Levels	VIP	P_value	Q_value	Fold_Change	Log_Fold change
Galactose	1	1.79	0.013	0.77	1.28	0.36
1,6-Anhydro-.beta.-D-glucose	2	1.38	0.013	0.77	1.41	0.49
Fructose	1	1.79	0.013	0.77	1.28	0.36
Glycylproline	1	1.37	0.042	0.77	1.39	0.48
Normetanephrine	1	2.11	0.022	0.77	1.64	0.71
S-Adenosylmethionine	1	2.04	0.012	0.77	1.27	0.35
Imidazoleacetic acid	1	1.84	0.033	0.77	1.27	0.35
Fructosamine	2	2.07	0.026	0.77	1.60	0.68
8-iso-15-Ketoprostaglandin F2.alpha.	2	1.02	0.028	0.77	0.77	-0.37
1,2-Di-(9Z,12Z,15Z-octadecatrienoyl)-sn-glycero-3-phosphocholine	2	1.80	0.002	0.77	1.29	0.37
Pro-Gly	1	1.37	0.042	0.77	1.39	0.48
L-Norvaline	2	1.32	0.032	0.77	1.20	0.27
Dimethylbenzyl_carbinyl_hexanoate	2	2.85	0.004	0.77	1.56	0.64
Methionine	1	1.89	0.010	0.77	1.28	0.35
Piperidine	2	1.42	0.004	0.77	1.41	0.49
22-Acetylpriverogenin_B	2	2.83	0.004	0.77	1.47	0.56
Ganoderiol_C	2	2.58	0.025	0.77	1.34	0.42
(3S,3'R,5R,6R)-7',8'-Didehydro-3,6-epoxy-5,6-dihydro-beta,beta-carotene-3',5-diol	2	1.07	0.047	0.77	1.79	0.84
trans-Resveratrol_4'-sulfate	2	1.81	0.012	0.77	1.39	0.48
Xylose	1	2.28	0.043	0.77	1.20	0.26
Fluvoxamine_acid	2	1.33	0.009	0.77	1.52	0.60
Hygric acid	1	2.10	0.001	0.77	0.65	-0.62
(1R,3R,4S,5R)-1,3,4 glucose -trihydroxy-5-[(E)-3-(4-hydroxy-3-methoxyphenyl)prop-2-enoyl]oxycyclohexane-1-carboxylic acid	2	1.29	0.039	0.77	1.22	0.29

Ectoine	1	1.78	0.008	0.77	1.37	0.46
1,5-Naphthalenediamine	2	1.25	0.044	0.77	1.74	0.80
Sorbose	1	1.79	0.013	0.77	1.28	0.36
9,12-Octadecadiynoic acid	2	2.38	0.043	0.77	1.23	0.30
Cystine	1	1.48	0.018	0.77	1.27	0.35
N-Formylglycine	2	1.99	0.044	0.77	1.28	0.35
