# Supplementary material for: Nontargeted metabolomics analysis of potential biomarkers for patients with chronic ischemic stroke in extremely cold rural regions: An exploratory case-control study
Source: PLoS One. 2026 Feb 20;21(2):e0341966. doi: 10.1371/journal.pone.0341966 (PMC12923066; doi:10.1371/journal.pone.0341966)
Supplement: S4 Table — (PDF) [file pone.0341966.s005.pdf]

S4 Table. The potential DEMs between Daur and Han patients with CIS

MS_name	Level	VIP	P_value	Q_value	Fold_Change	Log_Fold change
5,6-Dihydrouridine	1	2.58	0.005	0.72	1.29	0.37
Tauro-beta-muricholic acid	1	1.27	0.020	0.72	0.95	0.95
2-Aminooctanoic acid	1	2.13	0.046	0.72	0.65	-0.63
S-Adenosylmethionine	1	1.89	0.047	0.72	1.34	0.43
N-Phenylacetamide	1	2.14	0.012	0.72	1.53	0.62
Arabinono-1,4-lactone	1	1.68	0.027	0.72	1.48	0.57
Anabasine Hydrochloride	2	1.75	0.048	0.72	1.24	0.31
Cer(d18:1/16:0)	1	2.18	0.034	0.72	1.32	0.40
N4-Acetylcytidine	1	2.30	0.008	0.72	1.41	0.50
Heptadecanoyl_carnitine	2	1.93	0.009	0.72	1.76	0.81
PC(15:0/20:1(11Z))	2	2.13	0.011	0.72	1.69	0.76
PC(16:1(9Z)/14:0)	2	2.57	0.011	0.72	3.09	1.63
1-oleoyl-2-myristoyl-sn-glycero-3-phosphocholine	1	2.17	0.047	0.72	1.56	0.64
1,2-Di-(9Z,12Z,15Z-octadecatrienoyl)-sn-glycero-3-phosphocholine	2	2.22	0.008	0.72	1.33	0.41
PC(20:5(5Z,8Z,11Z,14Z,17Z)/15:0)	2	1.62	0.037	0.72	1.47	0.56
PG(18:1(9Z)/20:3(8Z,11Z,14Z))	2	2.29	0.003	0.72	1.40	0.49
LPE(P-16:0)	2	2.15	0.041	0.72	0.79	-0.35
1-Hexadecyl-2-(9Z-octadecenoyl)-sn-glycero-3-phosphoethanolamine	2	1.89	0.026	0.72	1.52	0.61
PE(20:3(8Z,11Z,14Z)/0:0)	2	1.97	0.031	0.72	1.35	0.43
LysoPE(22:4(7Z,10Z,13Z,16Z)/0:0)	2	2.08	0.039	0.72	2.60	1.38
LPC(17:0/0:0)	1	1.55	0.044	0.72	1.33	0.41
1-Stearoyl-2-arachidonoyl-sn-glycero-3-phosphoserine	2	2.97	0.001	0.72	0.49	-1.02
2-Oxooctanoic acid	2	2.12	0.035	0.72	0.64	-0.64

Phe-His	2	2.13	0.026	0.72	2.36	1.24
threo-Syringoylglycerol	2	1.73	0.041	0.72	2.19	1.13
2-Methylpropanamine	2	2.00	0.018	0.72	2.03	1.02
Tricin methyl ether	2	1.78	0.022	0.72	0.53	-0.90
N-Benzyladenine	2	1.84	0.029	0.72	1.45	0.54
2-Ethyl-5-methylpyridine	2	1.76	0.013	0.72	1.93	0.95
N-(5-Methyl-3-oxohexyl)alanine	2	2.16	0.006	0.72	2.46	1.30
1-(2-Pyrimidyl)piperazine	2	1.68	0.042	0.72	1.51	0.60
2-Aminonicotinic acid	1	1.76	0.045	0.72	1.39	0.48
3-Nitrophenylhydrazine	2	2.29	0.014	0.72	1.39	0.47
Corynanthin	2	2.03	0.025	0.72	2.14	1.09
Methyl paraoxon	2	2.71	0.009	0.72	729.61	9.51
Tributylamine	2	1.16	0.038	0.72	1.34	0.42
10-Methylundecanoic acid	2	1.81	0.048	0.72	1.40	0.49
